# Retention of foreign body in the gut can be a sign of congenital obstructive anomaly: a case report

**DOI:** 10.1186/1752-1947-2-293

**Published:** 2008-09-09

**Authors:** Pravas Chandra Subudhi, Shivaram Prasad Singh, Chudamani Meher, Omprakash Agrawal

**Affiliations:** 1 Department of Pediatric Surgery, SCB Medical College, Cuttack 753007, Orissa, India; 2 Department of Gastroenterology, SCB Medical College, Cuttack 753007, Orissa, India; 3 Beam Diagnostics, Cuttack 753001, Orissa, India

## Abstract

**Introduction:**

Small smooth objects that enter the gut nearly always pass uneventfully through the gastrointestinal tract. Retention of foreign objects may occur due to congenital obstructive anomaly of the gut.

**Case presentation:**

We report here a child who presented with features of small gut obstruction which were attributed to a foreign body impacted in the intestine. At surgery, an annular pancreas was detected and the foreign body was found to be lodged in the distended proximal duodenum.

**Conclusion:**

The reported case highlights the fact that an impacted radio-opaque foreign body in a child should warn the pediatrician to the possibility of an obstructive congenital anomaly.

## Introduction

Small round or oval objects that enter the stomach nearly always pass uneventfully through the gastrointestinal tract without requiring intervention. The retention of foreign objects within the duodenum is suggestive of partial obstruction, usually of congenital origin [1-3]. We describe a child presenting with features of high intestinal obstruction where retention of such an object led to the discovery of congenital duodenal stenosis producing partial obstruction.

## Case presentation

**Figure 1:**
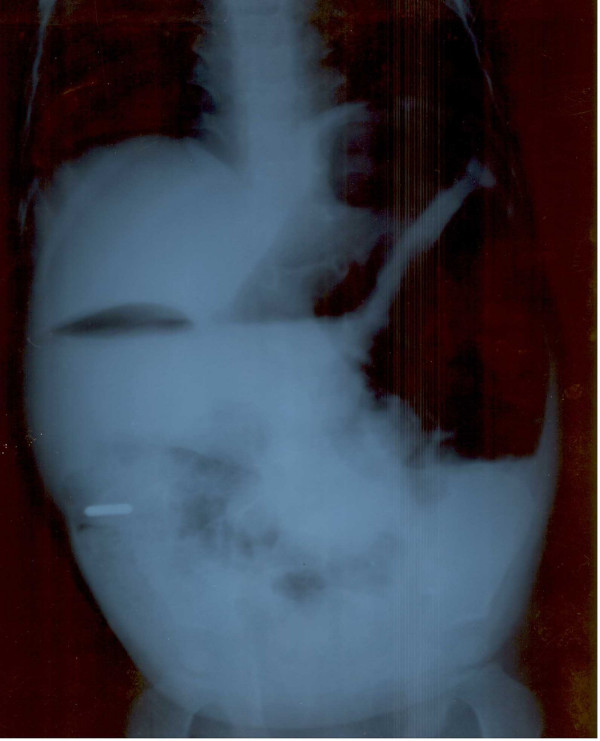
Plain radiograph of the abdomen showing the metallic foreign body in the right lower quadrant, the presence of a 'double bubble sign', and a few dilated loops of small bowel in the left upper quadrant.

A 32-month-old boy presented with a history of intermittent vomiting over the previous 15 months. The vomitus was generally non-bilious but occasionally bilious. The parents also noticed intermittent distension of his abdomen which subsided after vomiting. The symptoms seemed to commence after the child had swallowed a metallic pendant which was coin-shaped and about 12 mm in diameter; at the time of swallowing, the child was about 17 months old. He underwent repeated plain upright radiographs of the abdomen to localize the foreign body and to determine whether it had been passed. However, these continued to detect the foreign body. The last plain radiograph (Figure 1) of his abdomen showed the foreign body to be located in the right lower quadrant and it was surmised that the intestinal obstruction was due to impaction of the foreign body in the region of the terminal ileum. The child's parents were therefore advised that their child needed to undergo surgery for relief of the obstruction. However, a review of the plain upright radiograph of the abdomen showed the presence of a 'double bubble sign', in addition to a few dilated loops of small bowel in the left upper quadrant. A pre-operative diagnosis of duodenal obstruction was made with the possibility of another obstructive lesion in the small bowel. The foreign body was presumed to be lodged somewhere in the ileal loops. The child was then subjected to exploratory laparotomy. During surgery, his stomach and proximal duodenum were found to be grossly dilated with thickening of their walls, and an annular pancreas was detected encircling the second part of the duodenum. In addition, there was a membrane with a small aperture in the duodenum. Surprisingly, the metallic pendant was found lodged in the duodenum along with lot of debris including berry seeds. The third part of the duodenum was mobilized and duodenoduodenostomy was performed without dividing the pancreas.

## Discussion

Retention of elongated or pointed objects in the duodenum is a frequent problem. Long, sharp objects may perforate the duodenum and have been known to migrate widely in the abdomen. Early removal of such objects has been advised [4,5]. In addition, objects longer than 5 cm frequently fail to negotiate the C-curve and become impacted [5-7] and hence should be removed using an endoscope if possible. For blunt objects, some authors have also recommended intervention if the foreign body remains in the same location for more than a week [4,5].

Small round, oval, or cuboidal foreign objects nearly always pass through the gastrointestinal tract promptly, and stasis of such objects in the stomach or duodenum is extremely uncommon [1]. The retention of such foreign objects within the duodenum suggests partial obstruction, usually of congenital origin. In otherwise normal children, duodenal stenosis, prolapsing duodenal diaphragm, and annular pancreas may cause retention of swallowed foreign objects [1].

There are a few reports of radio-opaque foreign objects retained at the site of congenital duodenal obstruction [1-3]. Patients with duodenal stenosis alone or duodenal stenosis with annular pancreas may present with a variety of retained foreign materials in the stomach or proximal duodenum. Nuts, vegetable and fruit pits, and coins have been discovered at operation. Repeated abdominal roentgenograms should show that the foreign object is retained within the stomach or, more frequently, within the proximal duodenum. Upper gastrointestinal tract examination should confirm the presence of a duodenal anomaly. Duodenoduodenostomy or duodenojejunostomy should be performed after removal of the foreign object(s).

However, in spite of the persistence of the radio-opaque foreign body on plain X-rays of the abdomen, the possibility of an obstructing anomaly in this child was never considered. He continued to suffer for about 15 months until he was seen by a pediatric surgeon. However, even at the tertiary center, initially the surgeon and radiologists were confused by the location of the radio-opaque shadow in his right lower quadrant and a diagnosis of small gut obstruction was made; this was attributed to the foreign body being impacted in the intestine. However, during a review of the radiograph, the double bubble sign was appreciated and duodenal obstruction was suspected. At surgery, an annular pancreas was detected and the foreign body was found to be lodged in the distended proximal duodenum.

In adults, there are rare case reports of impaction by foreign bodies leading to detection of bowel stricture due to acquired diseases such as Crohn's disease [8,9]. However, in children with impaction or retention of foreign bodies, a congenital obstructing anomaly should always be kept in mind [1-3]. The case reported here was not subjected to proper investigations pre-operatively. In cases of radio-opaque foreign bodies, it is quite easy to follow the passage of the object periodically by plain abdominal radiography; however, this has limitations in studying bowel obstructions from foreign bodies which are not radio-opaque. Plain abdominal radiography has a sensitivity of 86% in the diagnosis of high-grade bowel obstruction and this will demonstrate air fluid levels with dilated small bowel loops [10,11]; an intramural width of small intestine of 3 cm or less is considered abnormal. An abdominal CT scan is of great help in diagnosing and detecting the etiology of intestinal obstruction in 73–95% of cases [10-12]. A CT scan may also be able to demonstrate the foreign body [8]. Generally, laparotomy is performed for diagnosis and management in cases of impacted foreign bodies in the gut. However, with increasing expertise, laparoscopy can be equally effective with all of the other advantages of a minimal access approach. Hence, laparoscopy is now increasingly being employed for removal of ingested foreign bodies impacted in the gastrointestinal tract [13,14].

## Conclusion

The present case is reported to highlight the fact that retention or non-passage of a radio-opaque foreign body in a child should alert the treating doctors to the possibility of an obstructive congenital anomaly.

## Consent

Written informed consent was obtained from the parents of the child for publication of this case report and accompanying image. A copy of the written consent is available for review by the Editor-in-Chief of this journal.

## Competing interests

The authors declare that they have no competing interests.

## Authors' contributions

SPS assessed and interpreted the patient's gastrointestinal symptoms and the investigations. CM and OA carried out the radiological examination while PCS performed the surgery on the child. All were major contributors in writing the manuscript and all authors read and approved the final manuscript.

## References

[B1] Kassner EG Rose JS Kottmeier PK Schneider M Gallow GM Retention of small foreign objects in the stomach and duodenum. A sign of partial obstruction caused by duodenal anomalies Radiology 1975 114 683 686 1118573 10.1148/114.3.683

[B2] Stanley P Law BS Young LW Down's syndrome, duodenal stenosis/annular pancreas, and a stack of coins Am J Dis Child 1988 142 459 460 2964775

[B3] Spitz I Management of ingested foreign bodies in childhood Br Med J 1971 4 5785 469 472 5125285 10.1136/bmj.4.5785.469 PMC1799648

[B4] Seo JK Endoscopic management of gastrointestinal foreign bodies in children Indian J Pediatr 1999 66 Suppl 1 S75 S80 11132474

[B5] Eisen GM Baron TH Domnitz JA Faigel DO Goldstein JL Johanson JF Mallery JS Raddawi HM VargoII JJ Waring JP Fanelli RD Harbough JW Guidelines for the management of ingested foreign bodies Gastrointest Endosc 2002 55 802 806 10.1016/S0016-5107(02)70407-0 12024131

[B6] Erbes J Babbitt DP Foreign bodies in the alimentary tract of infants and children Appl Ther 1965 7 1103 1109 5847723

[B7] Christie DL Ament ME Removal of foreign bodies from esophagus and stomach with flexible fiberoptic panendoscope Pediatrics 1976 57 931 934 934748

[B8] Slim R Chemaly M Yaghi C Honein K Moucari R Sayegh R Silent disease revealed by a fruit Gut 2006 55 181 10.1136/gut.2005.073270 16407383 PMC1856521

[B9] Amonkar SJ Hughes T Browell DA Crohn's disease discovered by an obstructing chick pea Br J Hosp Med (Lond) 2007 68 445 17847697

[B10] Lerma MA Mariscal JME Cordon FD Abril AG Oron EM Perez MJM Small bowel obstruction caused by Snail's shell: Radiographic and CT findings J Comput Assist Tomogr 2002 26 529 531 10.1097/00004728-200207000-00010 12218815

[B11] Maglinte DD Reyes BL Harmon BH Kelvin FM Turner WW Jr Hage JE Ng AC Chua GT Gage SN Reliability and role of plain film radiograph and CT in the diagnosis of small bowel obstruction AJR 1996 167 1451 1455 8956576 10.2214/ajr.167.6.8956576

[B12] Maglinte DD Balthazar EJ Kelvin FM Megibow AJ The role of radiology in the diagnosis of small bowel obstruction AJR 1997 168 1171 1180 9129407 10.2214/ajr.168.5.9129407

[B13] Chin EH Hazzan D Herron DM Salky B Laparoscopic retrieval of intraabdominal foreign bodies Surg Endosc 2007 21 1457 Epub 2007 May 19. 10.1007/s00464-006-9011-0 17593464

[B14] Palanivelu C Rangarajan M Rajapandian S Vittal SK Maheshkumaar GS Laparoscopic retrieval of 'stubborn' foreign bodies in the foregut: a case report and literature survey Surg Laparosc Endosc Percutan Tech 2007 17 528 531 18097316 10.1097/SLE.0b013e31815ac599

